# The Granuloma in Tuberculosis: Dynamics of a Host–Pathogen Collusion

**DOI:** 10.3389/fimmu.2012.00411

**Published:** 2013-01-07

**Authors:** Stefan Ehlers, Ulrich E. Schaible

**Affiliations:** ^1^Priority Research Area “Infections”, Research Center BorstelBorstel, Germany; ^2^Molecular Inflammation Medicine, Institute for Experimental Medicine, Christian-Albrechts-UniversityKiel, Germany; ^3^Department of Immunology, Faculty of Infectious and Tropical Medicine, London School of Hygiene and Tropical MedicineLondon, UK

**Keywords:** granuloma, tuberculosis, pulmonary, life cycle stages, immunopathology, evolution

## Abstract

A granuloma is defined as an inflammatory mononuclear cell infiltrate that, while capable of limiting growth of *Mycobacterium tuberculosis*, also provides a survival niche from which the bacteria may disseminate. The tuberculosis lesion is highly dynamic and shaped by both, immune response elements and the pathogen. In the granuloma, *M. tuberculosis* switches to a non-replicating but energy-generating life style whose detailed molecular characterization can identify novel targets for chemotherapy. To secure transmission to a new host, *M. tuberculosis* has evolved to drive T cell immunity to the point that necrotizing granulomas leak into bronchial cavities to facilitate expectoration of bacilli. From an evolutionary perspective it is therefore questionable whether vaccination and immunity enhancing strategies that merely mimic the natural immune response directed against *M. tuberculosis* infection can overcome pulmonary tuberculosis in the adult population. Juxtaposition of molecular pathology and immunology with microbial physiology and the use of novel imaging approaches afford an integrative view of the granuloma’s contribution to the life cycle of *M. tuberculosis*. This review revisits the different input of innate and adaptive immunity in granuloma biogenesis, with a focus on the co-evolutionary forces that redirect immune responses also to the benefit of the pathogen, i.e., its survival, propagation, and transmission.

## Introduction

The pathogenesis of tuberculosis used to be the investigative domain of two relatively separate, albeit complementary disciplines. On the one hand, molecular microbiologists decoded the survival strategies of *Mycobacterium tuberculosis* (*M. tuberculosis*), i.e., physiological and metabolic adaptation to the host environment, dynamics of replication, and synthesis and structures of the cell wall in order to define microbial factors important for virulence and persistence. On the other hand, infection immunologists analyzed innate and adaptive immune responses required to contain *M. tuberculosis* growth and dissemination, often welcoming the pathologist’s view on granuloma initiation, maintenance, and disintegration. In recent years, a more integrated view of tuberculosis pathogenesis has prevailed: the granuloma is not only recognized as a tissue reaction to limit bacillary growth and sequester infection but also as part of the successful life cycle of *M. tuberculosis*, thus representing the dynamic combat zone between both, the pathogen and host defense elements. This co-evolutionary perspective emphasizes the mutual shaping of the tissue microenvironment, which, at the same time, allows propagation and transmission of *M. tuberculosis*, yet restricts tissue damage to safeguard survival of the host. This review will highlight recent findings that have shifted the long-held paradigm that in TB the granuloma is primarily or uniquely relevant for protection of the host. This integrative model of the granuloma’s function in TB pathogenesis also challenges the concept that granulomas exclusively serve the interest of the pathogen.

## Incipient Granulomas: Fertile Soil for Mycobacterial Replication

After aerosol inhalation, the first host cell *M. tuberculosis* encounters is the alveolar macrophage, which phagocytoses but fails to kill the mycobacterial invaders, but does produce chemoattractants. Chemokines produced by alveolar macrophage and pneumocytes attract the first round of inflammatory cells, i.e., neutrophils, monocyte derived macrophages, NK cells, and γδ-T cells, which further promote inflammation and tissue remodeling (Feng et al., 2006; Lockhart et al., 2006; Eum et al., 2010). In mouse models of aerosol infection with mycobacteria, granuloma formation proceeds in the complete absence of specific immunity (North and Izzo, 1993; Hänsch et al., 1996; Smith et al., 1997). While TNF-α and IFN-γ accelerate inflammatory cell infiltration, they are not essential for granuloma initiation (Flynn et al., 1995; Smith et al., 1997). Non-activated macrophages, however, serve as feeder cells within the nascent granuloma (Davis and Ramakrishnan, 2009; Ehlers, 2010). Indeed, in the transparent zebrafish embryo model of *M. marinum* infection, a single virulence factor, which is present also in *M. tuberculosis*, ESAT6, is sufficient to induce matrix metalloprotease 9 production in epithelial cells (Volkman et al., 2010). This results in the recruitment of resting macrophages in which *M. marinum* replicates, and which even function as vectors that spread infection to other body tissues (Davis and Ramakrishnan, 2009). In sum, early innate responses to *M. tuberculosis* infection do little to restrict and much to promote *M. tuberculosis* replication. Consequently, a focal accumulation of mononuclear cells in various states of differentiation, i.e., the initial stage of a granuloma, is not *per se* protective. Therefore it is no big surprise that the lack of the innate Toll-like or NOD-like receptors in mice, though involved in recognition of mycobacteria and subsequent induction of inflammation, has no major impact on the course of aerosol *M. tuberculosis* infection (Gandotra et al., 2007; Reiling et al., 2007; Walter et al., 2010). Similarly, C-type lectins including mannose receptor, CD38, DC-SIGN, or MINCLE, all recognizing various mycobacterial cell wall glycolipids, do not contribute much to protection, most probably due to a high degree of redundancy between those receptors (Court et al., 2010; Behler et al., 2012; Heitmann et al., 2012). It should however be mentioned that the mycobacterial glycolipid ligand of Mincle, trehalose dimycolate, alone is sufficient to induce inflammation and granuloma-like structures upon injection into mice, which is diminished in Mincle KO mice (Geisel et al., 2005; Ishikawa et al., 2009; Schoenen et al., 2010; Lee et al., 2012). Trehalose dimycolate, a mycobacterial virulence factor on its own right, may therefore represent a driving force in granuloma formation.

## Mature Granulomas: Dynamic Heterogeneity

**Figure 1:**
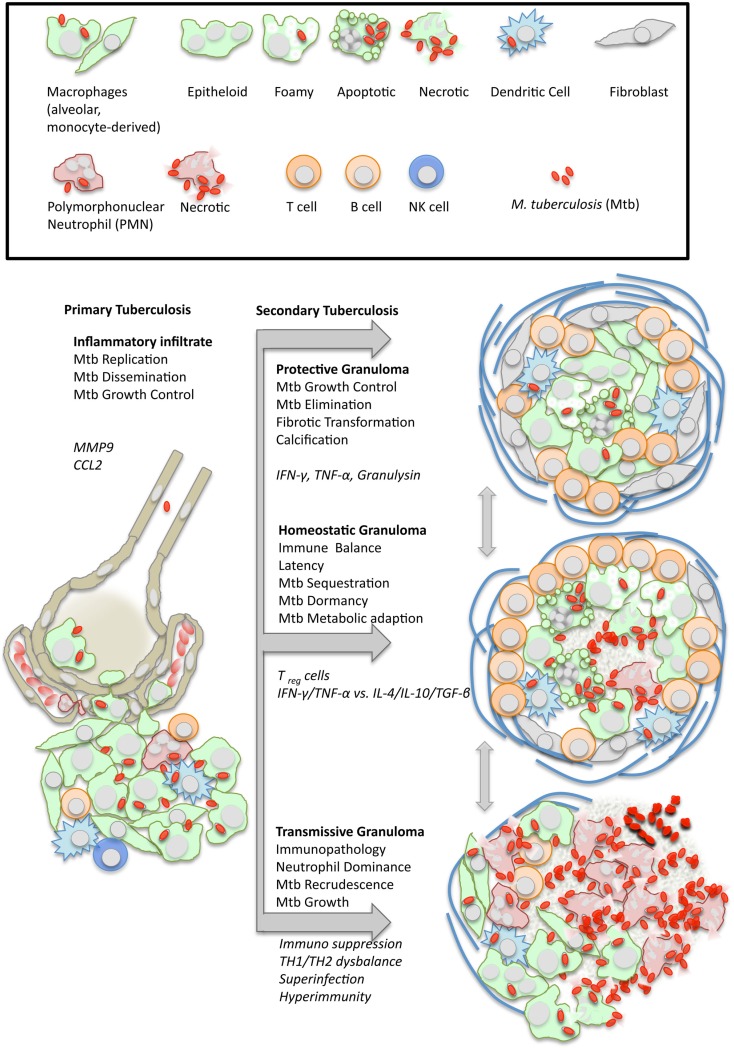
**Dynamics of granuloma formation and pathology in tuberculosis**. *M. tuberculosis* (Mtb) elicits a local inflammatory infiltrate which may give rise to (i) protective immunity, (ii) balanced inflammation (i.e., control of Mtb growth with little tissue damage), or (iii) endobronchial transmission following granuloma necrosis. The depicted types of organized granulomas are idealized and represent stages of a pathophysiological continuum. At the same time, they represent stages of the Mtb life cycle with either retarded growth or metabolic adaptation within the granulomatous lesion, or recrudescence and spreading to the next host following granuloma disruption. Italics indicate typical cellular and humoral mediators involved in granuloma differentiation which are addressed in more detail in the text.

Following migration from the initial inflammatory focus to the regional lymph nodes, dendritic cells prime T cells for differentiation into predominantly TH1 and TH17 as well as cytotoxic T effector cells (Cooper, 2009). Recirculating primed pathogen specific T cells are critical for activating macrophages and curtail mycobacterial growth within the nascent granulomatous lesion (North and Jung, 2004). In the presence of activated T cells, the granuloma becomes fully organized, with mycobacteria-harboring macrophages at the center surrounded by a rim of lymphocytes. The ensuing stale-mate between host and pathogen, however, is much more dynamic than previously thought, and involves continuous loss of cells by cell death and replenishment by cellular recruitment, as well as vascular and tissue remodeling (Figure 1). Apart from inflammatory immune cells also mesenchymal stem cells are recruited, which seem to promote infection (Raghuvanshi et al., 2010). Pathologists have long been enamored with describing tuberculosis lesions as “exsudative, suppurative, miliary, caseous, circumscribed, fibro-calcified,” and so on. A relatively recent analysis of lung lesions removed by surgery focused on two highly disparate structures, cavitary lesions and tuberculomas (Ulrichs and Kaufmann, 2006). Cavities were surrounded by few vessels and a diffuse pattern of proliferating cells, while tuberculomas exhibited a highly organized framework of follicle-like structures and high vascularization.

By describing variety and heterogeneity, pathologists have often tried to place different types of lesions in a seemingly logical temporal sequence to one another, insinuating developmental relationships (Dannenberg and Rook, 1994). Using sophisticated *in vivo* imaging reporter technology with molecular probes detecting host tissue metabolism as a measure for inflammatory activity, scientists are currently re-discovering this enormous heterogeneity of tuberculosis lesions, with the pioneers’ advantage of following the same granuloma over time (Barry et al., 2009). Thus, “centro-acinary, perifocal, tree-in-bud, metabolically active” have become useful phenotypic labels identifying several distinct types of lesions co-existing within a single individual. More surprisingly, these lesions may independently regress, and even vanish over time, while others flourish and exacerbate even under treatment (Barry et al., 2009; Lin and Flynn, 2010). There does not appear to be a clear linear relationship between these lesions, but rather a continuous spectrum. Macrophages within infiltrates form a scaffold to direct the movement of lymphocytes in search of antigenic stimulation (Egen et al., 2008, 2011), but both, a delay in T cell arrival as well as inhibition of T cell responses within the lesion account for a heterogeneous antimicrobial response and persistence of tubercle bacilli.

One theory contends that, even during latency, macrophages from time to time egress from the lesion and spread the infection to other parts of the lung (Cardona and Ruiz-Manzano, 2004; Cardona, 2009). Although this defies the very definition of a latent infection since migration of infective macrophages through the airspaces would effectively make this latent infection a patent one, it may reflect the flaring-up of quiescent lesions during apparent “latency.” A consensus is emerging that not only active, but also latent disease shows a spectrum of lesional activity (Lin et al., 2009). In this respect, *in vivo* imaging may for the first time deliver functional classifications of diverse “latency” stages, superseding the current rather static description of lasting immune responsiveness to an infectious stimulus that occurred in the past (Mack et al., 2009). It would be interesting to apply the imaging approach to screen clinically healthy individuals in high-transmission populations for incipient active lesions. Therapeutic consequences are evident, in that the identification of functional biomarkers indicating latent infections would permit specific preventive chemotherapy of those individuals at greatest risk for reactivating tuberculosis (Russell et al., 2010). Interferon-α and neutrophil driven transcriptome signatures have recently been described as markers of susceptibility to active human tuberculosis (Berry et al., 2010). Experimentally, the absence of IFN-αβ signaling in mice improved outcome after infection with highly virulent but not with attenuated (Cooper et al., 2000; Manca et al., 2001), strains of *M. tuberculosis*, and genetically susceptible mouse strains survived longer when neutrophils were depleted (Keller et al., 2006). Whether these signatures can serve as prospective susceptibility markers for preventive treatment of latent tuberculosis patients has to be proven in longitudinal studies in high-transmission populations (Berry et al., 2010). One caveat is that some of these “biomarkers” may not be entirely specific for tuberculosis, but rather indicative of other chronic granulomatous conditions such as sarcoidosis (Maertzdorf et al., 2012).

A number of animal model systems, ranging from mice, guinea pigs, rabbits, minipigs, to marmoset, cynomolgous, and macaque non-human primates, are in use to depict various aspects of granuloma immunopathology. No single model system is perfect, but they all reflect individual aspects of human tuberculosis and, if taken with a grain of salt, contribute to stratifying the complexity of pathology in humans, which cannot yet be examined directly in molecular detail at the lesional site in humans. What has become most evident from analyzing the diversity of granulomas is that no cross-sectional or even systemic marker can adequately represent the succinctly local microenvironments of pulmonary granulomas. Therefore, biomarker development is facing a formidable challenge!

## Versatile Metabolism Within Granulomas: Non-Replicating Persistence of *M. tuberculosis*

Gluconeogenesis is necessary for *M. tuberculosis* throughout the infection, i.e., not only in the phase of persistence, and phosphoenolpyruvate kinase is the critical gate enzyme (Marrero et al., 2010). Within the granuloma, it is assumed that *M. tuberculosis* replicates only very little, but remains fully capable of generating energy (see Figure 1). Genome-wide expression profiling of *M. tuberculosis* RNA isolated from chronically infected mouse lungs or sputum of tuberculosis patients revealed transcriptional signatures reflective of environmental conditions such as low pH, oxygen depletion, iron limitation, nitrosative stress, and nutrient starvation (Timm et al., 2003; Voskuil et al., 2004; Talaat et al., 2007; Garton et al., 2008). This explains the attenuated phenotype of mutant *M. tuberculosis* defective in the leucin, lysine, purine, or pantothenate biosynthetic pathways, or which are deficient in iron acquisition or the stringent response (Boshoff and Barry, 2005).

The DosR regulon is a genetic program that critically governs survival in the absence of respiration (Leistikow et al., 2010). The conserved presence of long sequences of the DosR regulon in environmental mycobacteria suggests that it did not evolve specifically for survival within the mammalian host. Instead, the regulon may have evolved to cope with conditions within the environment such as very low oxygen tension. Thus, in a hypothetical ancestral *M. tuberculosis* strain, DosR gene products may have allowed the bacteria to survive in a specific niche in a non-replicating state, ensuring positive selection (Bartek et al., 2009).

Using a dual staining approach, three *M. tuberculosis* subpopulations were found in hypoxic culture and in lung sections of mice and guinea pigs. Bacteria were either exclusively acid-fast positive, exclusively immunofluorescent positive or acid-fast and immunofluorescent positive (Ryan et al., 2010). These results suggest that *M. tuberculosis* exists as multiple populations with distinct cell wall properties even within a seemingly single microenvironment, advocating the development of analytical tools at the single cell level. Indeed, intracellular mycobacteria have altered cell wall lipid pattern as those grown in broth (Fischer et al., 2001). Evidence from guinea pigs treated with antibiotics indicates that *M. tuberculosis* may persist extracellularly within biofilm-like structures that consist of DNA and neutrophil debris, in a hypoxic and iron-rich environment with incomplete dystrophic calcification (Lenaerts et al., 2007). These biofilms appear similar to those shown for biofilms of another lung pathogen, *Pseudomonas aeruginosa* (Parks et al., 2009). These niches where access of antibiotics is likely compromised may serve as the primary sites of disease reactivation, and mimicking these tissue conditions in axenic culture will be essential for successful *in vitro* compound screening for next generation anti-mycobacterials.

Even when the drugs reach the bacteria, the actual mechanism of killing *M. tuberculosis* remains unclear for most anti-tuberculosis drugs. A case in point is isoniazid. It is generally assumed that INH is only effective on actively replicating *M. tuberculosis*. The fact that INH preventive chemotherapy can reduce the level of tuberculosis manifestation in individuals diagnosed with latent infection has been a major argument for the contention that quiescent tuberculosis lesions contain actively dividing *M. tuberculosis* (Diel et al., 2008). It is clear that INH, following its activation by the catalase peroxidase KatG, inhibits mycolic acid synthesis. More specifically, an INH-NAD adduct inhibits the fatty acid synthase II enoyl-ACP reductase InhA, leading to the accumulation of long-chain fatty acids (Vilcheze and Jacobs, 2007). How exactly this precipitates *M. tuberculosis* death, however, is the subject of ongoing investigations at the single cell level within calibrated fermentation chambers, using fluorescent reporter probes (Golchin et al., 2012).

Detailing the mechanisms and kinetics of how drugs kill *M. tuberculosis* *in vitro* will be a crucial initial step for improving anti-tuberculosis drug efficacy. An even more challenging task will be to define and refine how drugs penetrate and work effectively within tuberculosis lesions. This requires full knowledge of the tissue microenvironment influenced by both, mycobacteria as well as host responses, and includes micronutrient availability for both “partners,” defense cells and microbe. Innovative imaging techniques paired with fluorescent or luminescent reporter strains of *M. tuberculosis* are important tools for monitoring pathogenesis *in situ* and drug efficacy testing (Andreu et al., 2010; Carroll et al., 2010; Kong et al., 2010; Zelmer et al., 2012). Of even greater interest will be *M. tuberculosis* sensor strains, which can report physico-chemical conditions *in situ* such as low iron concentration or drug exposure.

How does *M. tuberculosis* exit the state of seeming dormancy and resume growth? It may take one cue from the production of resuscitation promoting factors (Rpf), RpfB being the most relevant isoform out of five encoded in the *M. tuberculosis* genome based on persistence assays with targeted deletion mutants (Kana et al., 2008; see Figure 1). The structure of RpfB contains peculiar features that are also shared by G5 domains involved in biofilm formation (Ruggiero et al., 2009), providing a further link to evolutionarily conserved pathways of adaptation to adverse environmental conditions by changing growth patterns.

Foamy macrophages are key constituents of tuberculosis lesions, representing macrophages packed with lipid bodies following activation via Toll-like receptors and proinflammatory signals (D’Avila et al., 2006). These lipid bodies are the consequence of an imbalance between the influx and efflux of low-density lipoprotein particles from the serum. The phenotype can be induced *in vitro*, in peripheral blood cells incubated with distinct mycobacteria-derived oxygenated ketomycolic and hydroxyl-mycolic acids (Peyron et al., 2008). Lipid bodies may provide ideal nutrition for a bacterium that is changing its metabolism to digest fatty acids, as evidenced by upregulation of the gating enzyme of the glyoxylate shunt, isocitrate lyase, and of the methylcitrate pathway, methylcitrate dehydratase, in the intracellular life stage (McKinney et al., 2000; Gould et al., 2006; Mattow et al., 2006; Munoz-Elias et al., 2006).

*Mycobacterium tuberculosis* has also been shown to metabolize cholesterol (Pandey and Sassetti, 2008), and several genes such as Mce4, HsaC, and Icl1, which were previously linked to propionate metabolism, may be functionally linked through cholesterol degradation. Cholesterol metabolism requires altering of transcription and metabolic profiles of *M. tuberculosis* to access propionyl-CoA and pyruvate pools through the methylcitrate cycle. Consequently, gene deletion mutants therein are handicapped for intracellular growth (Griffin et al., 2012). Analysis of lipids from the caseum of human tuberculosis granulomas revealed that the main lipid species are cholesterol, cholesteryl ester, triacylglycerides, and lactosylceramide, implicating low-density lipoprotein-derived lipids as the most likely source (Kim et al., 2010). Granuloma necrosis, at least in mouse *M. tuberculosis* or *M. avium* infection, is often associated with high bacterial burden, and virulent mycobacteria can inhibit membrane repair, which causes necrosis in infected macrophages (Divangahi et al., 2009). A conceivable scenario, therefore, is that the death of foamy macrophages results in the accumulation of lipid debris making up the caseum at the center of the granuloma (Russell et al., 2009; Behar et al., 2010). Other cells may also contribute to cellular debris as neutrophils represent a major cell population in TB lesions, which has an exquisitely high death toll due to their short half-life and *M. tuberculosis* driven necrosis (Eum et al., 2010; Corleis et al., 2012; see Figure 1).

The initial necrotic nidus may be induced in the absence of any adaptive immune response, as guinea pigs exhibit focal necrosis within granulomas as early as 10 days after infection (Turner et al., 2003). Overwhelming experimental and clinical evidence, however, suggests that promotion of full-fledged central caseation in mycobacterial granulomas requires a hypersensitivity reaction, precipitated by CD4+ T cells faced with a high antigenic load (Dannenberg, 1991; Ehlers et al., 2001; Sanghi et al., 2011).

## Preparing for the Exit: Granuloma Necrosis as a Transmission Strategy

There is much debate about whether tissue necrosis begins with central necrotization of preformed granulomas, or whether caseation and cavitation develop from so-called lipid pneumonia accompanying regrowth of *M. tuberculosis* persisting outside of granulomas (e.g., in epithelial cells, adipocytes, etc.; Hernandez-Pando et al., 2000; Neyrolles et al., 2006). Evidence from the former comes from studies in rabbits, guinea pigs, and designer mouse models, while evidence for the latter is derived from histopathological studies in men and mice. For example, mice bearing the sst-susceptible allele develop centrally necrotizing pulmonary granulomas in which *M. tuberculosis* growth is rampant. The host resistance gene, Ipr1, encoded within the sst1 locus, regulates the infected macrophages’ mechanism of death (Kramnik, 2008). In dermally infected NOS2-deficient mice treated with a neutralizing anti-IFN-γ antibody, established granulomas centrally necrotize, showing signs of hypoxia and abundant cathepsin G activity (Reece et al., 2010). In guinea pigs, necrosis within granulomatous lesions can occur even before a robust T cell response has developed (Turner et al., 2003). In rabbits, cavitation has generally been attributed to a strong DTH response to *M. bovis* infection precipitating caseation, cavitation, and liquefaction of caseum within circumscript granulomas (Dannenberg and Sugimoto, 1976; Dannenberg and Collins, 2001).

By examining a histological archive of tuberculosis lesions in humans and revisiting the Cornell model of reactivation tuberculosis in mice, Hunter et al. (2007) came to the conclusion that reactivation of *M. tuberculosis* infection begins as a lipid pneumonia with bronchial obstruction and does not start from disintegrating granulomas. A very potent component of *M. tuberculosis* to which this necrosis-inducing activity is attributed is cord factor, or trehalose dimycolate which can induce inflammatory infiltrates including foam cell formation but is also a virulence factor promoting intracellular survival (Hunter et al., 2006; Kim et al., 2010). Taken together, caseation appears to correlate with pathogen-mediated dysregulation of host lipid metabolism.

As neutrophils represent the predominant cell population in broncho-alveolar lavages of active tuberculosis patients they may be more relevant in transmission of *M. tuberculosis* than in controlling infection (Eum et al., 2010). Considerable controversy exists over whether neutrophils are able to kill mycobacteria and a recent review on the issue concluded that these otherwise potent anti-bacterial effector cells fail to eliminate *M. tuberculosis* (Korbel et al., 2008). Indeed, virulent *M. tuberculosis* quickly cause necrotic cell death of human neutrophils upon infection *in vitro* by inducing reactive oxygen intermediates (ROI) allowing the mycobacteria to escape neutrophil-mediated killing. In contrast, an attenuated *M. tuberculosis* mutant lacking the virulence associated RD1 region fails to induce necrosis and falls victim to neutrophils’ armamentarium, i.e., ROI (Corleis et al., 2012). More importantly, invading neutrophils with their payload of cytotoxic molecules may cause substantial tissue destruction and remodeling. It can be envisaged that misguided but anti-microbially ineffective neutrophils invade existing (latent?) tuberculosis lesions and prompt the immunopathological cascade toward active lesions, providing further host cell lipid substrate for *M. tuberculosis* growth and biofilm formation to secure subsequent transmission. Here, super-infection with a novel *M. tuberculosis* strain or even another unrelated co-infecting lung pathogen may represent an as yet under-appreciated cause of reactivation (see Figure 1). In addition, systemic co-infections of *M. tuberculosis* and unrelated pathogens beyond HIV may also modulate lung immunity in the latently infected. This was recently demonstrated in the murine model of malaria-associated acute respiratory distress syndrome, in which the mycobacterial burden was exacerbated in malaria – *M. tuberculosis* co-infected mice (Mueller et al., 2012).

Sequencing the genomes of 21 *M. tuberculosis* strains representative of the global diversity identified very little sequence variation in 491 experimentally confirmed human T cell epitopes, indicating purifying selection (Comas et al., 2010). This finding led to the hypothesis that *M. tuberculosis* benefits from recognition by human T cells. In view of its confinement to the granuloma, *M. tuberculosis* may have devised an exit strategy that exploits T cell activation. TH1 responses (IFN-γ, IRF-1) were shown to drive granuloma necrosis in a model of mycobacteria-induced pulmonary immunopathology (Ehlers et al., 2001; Aly et al., 2009), and TH2 responses have also been associated with increased cavity formation and tissue destruction in humans and mice (Rook, 2007); Hölscher, C., personal communication). Cavity formation in rabbits, monkeys, and man are clearly results of a long-lasting T cell-driven immune activation, low numbers of CD4+ cells virtually precluding granuloma necrosis (Chaisson et al., 1987; Capuano et al., 2003). Moreover, AIDS patients with tuberculosis have a different pathology and often, their sputum is negative for acid-fast bacteria, further suggesting that transmission-facilitating granulomas are immune driven (Aaron et al., 2004, see Figure 1).

An integrated view of current findings would hold that individual components of *M. tuberculosis*, such as trehalose dimycolate, allow for foam cell formation and macrophage necrosis, but full-blown central necrosis of an established granuloma requires T cell participation, either in the form of a hyperactive IFN-γ production or a superimposed IL-4/IL-13 response. The latter induces alternative macrophage activation (Gordon and Martinez, 2010), and arginase 1 is a potential biomarker for reactivation tuberculosis and granulomas prone to necrotizing. Neutrophils, which are also potent producers of arginase 1, may even amplify this immunopathogenic cascade.

Are the “fat and lazy” persister bacilli present in the sputum of cavitary patients specifically adapted for transmission (Garton et al., 2008)? Very little is known about the physical parameters in individual *M. tuberculosis* bacteria relevant for aerosolization, survival in the environment, or infectivity (i.e., inoculum “take” in the alveolar macrophage; Fennelly et al., 2004). Also, there is a growing debate as to whether low dose aerosol infection adequately reflects the situation in endemic countries where exposure to putatively larger numbers of coughed-up *M. tuberculosis* may be the crucial factor overriding naturally existing or vaccine-induced immunity (Rook et al., 2009).

## The Bottom Line: Does *M. tuberculosis* Universally Profit from Granuloma Formation?

Given the recent focus on the stunning discovery that *M. tuberculosis* may direct granuloma induction and maturation as part of its life cycle, the fact that, in the absence of granuloma formation there is no protection, at least in the human host, has been unduly neglected. Granulomas afford a unique juxtaposition of activating T cells and mycobacteria-laden macrophages, and the temporal coincidence of T cell differentiation, granuloma consolidation, and reduction of *M. tuberculosis* growth in all animal models that mimic human disease suggests causality (North and Jung, 2004). It is important to emphasize that T cell memory affords 10-fold lower bacterial loads in infected organs of vaccinated animals, and that the majority of bacteria reside within granulomas and not randomly distributed throughout the body. Therefore, while a mycobacteria-focused view on granulomas was long overdue (Russell, 2007; Ramakrishnan, 2012), this should by no means neutralize the evidence in favor of the protective infection-sequestrating granuloma:

– T cells transfer protective immunity and granuloma formation (Orme and Collins, 1983).
– HIV-infected individuals exhibit poor granulomatous inflammation and poor control of mycobacterial growth (Lawn et al., 2002).
– Macrophage accumulations (or innate granulomas) do not efficiently kill mycobacteria unless activated by T cells (North and Izzo, 1993; Smith et al., 1997).
– Interferon-gamma provided by NK cells alone is insufficient to provide full protection in the absence of T cells (Feng et al., 2006).

It is certainly true that the host-centric view has prevailed for too long, neglecting the important input of *M. tuberculosis* in shaping the granuloma to its advantage, but it is unwise to underestimate the power of T cell-mediated macrophage activation, which takes place at the site of granulomatous inflammation, as it currently provides the only venue for preventive vaccination strategies.

## Conclusion: Is It Possible to be Better than Nature?

One of the implications of the integrative view on *M. tuberculosis*’ life cycle embraced here is that, to stop *M. tuberculosis* from multiplying and transmitting, simple imitation, or augmentation of the natural host response to infection is likely to fail. Unless T cells can be trained to recognize *M. tuberculosis* as soon as it enters the alveolar macrophage, one of the best vaccination strategies might be to bypass the regulatory networks *M. tuberculosis* itself initiates to establish its niche for replication. If anything, vaccines would have to mitigate TH1 and TH2 responses and altogether blunt regulatory T cell responses to allow more protective immunity while avoiding damaging pathology (Rook et al., 2005). This may be impossible to achieve purely by vaccination, leaving ample opportunity for adjunct immunomodulatory measures. Indeed, inhibition of inflammation may prove to be a superior adjunct strategy to improve the outcome of antibiotic therapy (Churchyard et al., 2009). For example, blockade of PDE4 together with INH shortened the duration of treatment by one month and reduced pathology in a rabbit model of tuberculosis (Subbian et al., 2011). During the Immune Reconstitution Inflammatory Syndrome (IRIS), which occurs in *M. tuberculosis*-infected AIDS patients receiving highly active antiretroviral therapy, corticosteroid therapy is highly effective. However, it does not reproducibly interfere with TH1 responses but reduces granzyme B and perforin levels, indicating an involvement of CD8+ T or NK cells in inflammatory exacerbation (Meintjes et al., 2009).

A potential problem with current immunization strategies against tuberculosis might be that they prime an immune response to epitopes that have been highly conserved in *M. tuberculosis*, because these dominant T cell targets ensure escape from the granuloma and thus transmission. Indeed, in an alternative strategy that refocused the T cell response to specificities that are not normally recognized during natural infection, a more efficient protection against murine *M. tuberculosis* infection was induced than afforded by immunization with the immunodominant epitope (Aagaard et al., 2009). This increased efficacy was associated with elevated numbers of multifunctional T cells, producing TNF, IFN-γ, and IL-2 at high levels.

Further considerations for improving vaccines take into account that *M. tuberculosis* responds to the host immune response by regulating and diversifying its own gene expression; for example, during latency, stage-specific antigens are expressed that represent its metabolic adaptation and can effectively be utilized to discriminate, by immunodiagnosis of host T cell responses against these antigens, between acute and latent infection (Demissie et al., 2006). Based on these findings, a novel subunit combination vaccine was developed: H56, comprising three *M. tuberculosis* antigens, which are expressed either early in infection (Ag85, ESAT6) or during the latent phase (Rv2660c), not only boosts BCG-primed immunity but also induces multifunctional T cell-mediated immune protection on its own before and after exposure to *M. tuberculosis* (Aagaard et al., 2011). Ag85 is expressed by both, BCG and *M. tuberculosis*, and is therefore responsible for the boost effect, whereas ESAT6 is exclusive for the latter one and expressed mostly during early, active replication stages. Rv2660c however, is expressed during the entire course of infection in mice (even by starved and dormant *M. tuberculosis*) but is only a weak IFN-γ inducer by itself. It becomes mildly immunogenic only when fused to Ag85-ESAT6 thereby obviating immune-mediated exacerbation of the disease in infected individuals. In sum, the selection and combination of antigens and epitopes specific for different stages of TB may help outwit *M. tuberculosis* and control reactivation.

It would be unreasonable to call the TB granuloma an unsuccessful host defence, as it successfully contains the infectious focus in more than 90% of cases. The 10% of individuals that progress toward TB disease suffer from a disbalanced inflammatory reaction, be it due to too little innate or adaptive immunity or due to unrestrained hypersensitivity reactions. There is no balance without a trade-off: in the case of TB this is the Janus face of T cell immunity (which can be detrimental when overzealous). Any intervention thus must be regulatory in nature rather than proinflammatory, and the rational design of therapies and vaccines must take this into account.

## Conflict of Interest Statement

The authors declare that the research was conducted in the absence of any commercial or financial relationships that could be construed as a potential conflict of interest.
